# Solar Cell Based on Hybrid Structural SiNW/Poly(3,4 ethylenedioxythiophene): Poly(styrenesulfonate)/Graphene

**DOI:** 10.1002/gch2.202000010

**Published:** 2020-05-17

**Authors:** Nguyen Ngoc Anh, Nguyen Van Chuc, Bui Hung Thang, Pham Van Nhat, NguyenVan Hao, Doan Dinh Phuong, Phan Ngoc Minh, Thiyagu Subramani, Naoki Fukata, Pham Van Trinh

**Affiliations:** ^1^ Institute of Materials Science Vietnam Academy of Science and Technology 18 Hoang Quoc Viet Str., Cau Giay Hanoi 10000 Vietnam; ^2^ Graduate University of Science and Technology Vietnam Academy of Science and Technology 18 Hoang Quoc Viet Str., Cau Giay Hanoi 10000 Vietnam; ^3^ University of Science and Technology of Hanoi Vietnam Academy of Science and Technology 18 Hoang Quoc Viet Str., Cau Giay Hanoi 10000 Vietnam; ^4^ Faculty of Physics and Technology TNU‐University of Sciences Tan Thinh Ward Thai Nguyen 24000 Vietnam; ^5^ International Center for Materials Nanoarchitectonics National Institute for Materials Science 1‐1 Namiki Tsukuba Ibaraki 305‐0044 Japan

**Keywords:** conversion efficiencies, hybrid solar cells, PEDOT:PSS, silicon nanowires

## Abstract

Solar energy is considered as a potential alternative energy source. The solar cell is classified into three main types: i) solar cells based on bulk silicon materials (monocrystalline, polycrystalline), ii) thin‐film solar cells (CIGS, CdTe, DSSC, etc.), and iii) solar cells based on nanostructures and nanomaterials. Nowadays, commercial solar cells are usually made by bulk silicon material, which requires not only high fabrication costs but also limited performance. In this study, the fabrication of high‐performance solar cells based on hybrid structure of silicon nanowires/poly(3,4‐ethylenedioxythiophene):poly(styrenesulfonate)/graphene (SiNW/PEDOT:PSS/Gr) is focused upon. SiNWs with different lengths of 125, 400, 800 nm, and 2 µm are fabricated by a metal‐assisted chemical etching method, and their influence on the performance of the hybrid solar cells is studied and investigated. The experimental results indicate that the suitable SiNW length for the fabrication of the hybrid solar cells is about 400 nm and the best power conversion efficiency obtained is about 9.05%, which is about 2.1 times higher than that of the planar Si solar cell.

## Graphical abstract

Hybrid solar cells based on silicon nanowire (SiNW)/poly(3,4‐ethylenedioxythiophene):poly(styrenesulfonate)/graphene are fabricated and investigated. The highest power conversion efficiency obtained is 9.05% for the solar cell using 400 nm SiNWs, which is nearly 2.1 times higher compared to the planar Si solar cell. The enhancement is attributed to low reflection, strong broadband light absorption, and efficient charge‐carrier collection of SiNWs.

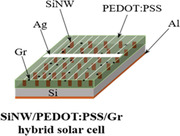

## 1 Introduction

The solar cells based on nanostructured materials have been received much attention from scientists in improving the power conversion efficiency.^[^ ^1^, ^2^ ^]^ Improving light trapping and photocarrier collection is one of the most promising methods to enhance the power conversion efficiency (PCE) of the solar cells. Among all the structures, silicon nanowires (SiNWs) structure not only exhibits an excellent absorption capacity but also provides a large surface area in comparison to bulk Si or thin‐film Si.^[^ ^3^, ^4^, ^5^, ^6^ ^]^ Therefore, using the SiNW structure for the fabrication of solar cells have been considering as a potential technique to improve the conversion efficiency and reduce cost production in comparison with solar cell based on thin film and bulk Si structures.^[^ ^3^ ^]^ Garnett and Yang have studied solar cells using SiNW to increase the area of the p–n junction layer based on the core–shell structure. The photoelectric conversion efficiency obtained was 0.5%.^[^ ^7^ ^]^ Ko et al. develop a solar cell based on SiNW asymmetric structure. The results show that the reflectivity of solar cells is very low (around 4–5%) and the photoelectric conversion efficiency is ≈8%.^[^ ^8^ ^]^ However, this type of solar cells is expensive because the manufacturing process requires expensive equipment and complex processes, such as high temperature and high vacuum conditions. Therefore, a new type of solar cell has been studied which is based on hybrid structures combining SiNWs and organic materials.^[^ ^9^, ^10^, ^11^, ^12^, ^13^ ^]^ This type of solar cell has low cost, lightweight and high flexibility.^[^ ^12^, ^13^ ^]^ Among all the organic materials, poly(3,4‐ethylene dioxythiophene):poly(styrene sulfonate) (PEDOT:PSS) is widely used in the fabrication of solar cells based on SiNW/organic structure.^[^ ^14^, ^15^, ^16^, ^17^, ^18^, ^19^ ^]^ Recently, several studies have been carried out to improve the working stability of solar cell with temperature, humidity, and reduce the degradation of organic materials as well as increase the uniform of a conductive polymer layer on the Si substrate by employing carbon nanotubes, fullerenes, graphene oxide as additive materials.^[^ ^20^, ^21^, ^22^, ^23^, ^24^, ^25^, ^26^, ^27^ ^]^ Graphene has excellent properties such as low impedance, high transmittance, good mechanical properties, high thermal stability, and chemical stability. In addition, graphene easy to functionalized with various functional groups, which can combine easily with organic and/or inorganic materials.^[^ ^28^ ^]^ Moreover, graphene could be fabricated with low‐cost production and large scale.^[^ ^29^ ^]^ Therefore, graphene can be used as a transparent electrode, electron transport layer, hole transport layer, or electron/hole separation layer combined with conductive polymers in fabricating the solar cells based on organic/inorganic hybrid structure. In this study, SiNWs fabricated with different lengths of 125, 400, 800 nm, and 2 µm by metal‐assisted chemical etching method were used for hybrid solar cell fabrication. The influence of SiNW length on the optical properties and PCE of the solar cells was investigated and presented.

## 2 Results and Discussion

**Figure 1:**
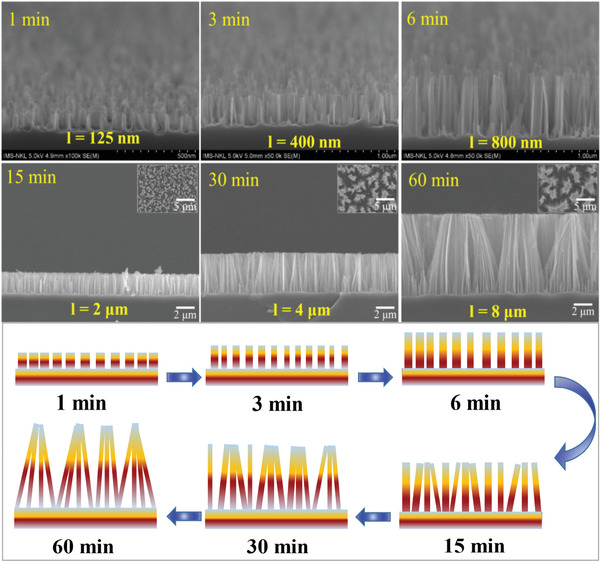
SEM image of SiNW prepared by chemical etching and schematic view of the morphology of SiNW with different etching time.

**Figure** **1** shows the scanning electron microscopic (SEM) images of SiNW structures with different etching times. The obtained results show that the increase of the etching time will lead to an increase the length of SiNWs. The SiNWs formed vertically to the silicon substrates with high density and diameter in the range of 100–200 nm. The etching rate of SiNW structure was determined to be 133 nm min^−1^. The obtained results show that SiNW length can be controlled by changing the etching time. It is interesting to note that the etching time seems not much influence to the size and shape of the SiNWs. Besides, no trapezoid shape and smaller size of SiNWs were observed. This demonstrated that the sidewall etching can be neglected in the formation of SiNWs. This is consistent with the other work reported recently.^[^ ^30^ ^]^ However, the congregation of the tips of the SiNWs has observed with the samples etched longer than 6 min (Figure 1). In other words, the aggregation of tips will happen if the length of SiNWs is long enough. The formation of the tip aggregation may have restricted the etching agent, thus decreased the etching rate over a long time. Besides, this cluster may prevent the penetration of PEDOT:PSS/Gr solution into the SiNW structure during the hybrid solar cell fabrication process. The influence of the SiNW length on the optical properties and the performance of the hybrid solar cells based on SiNW/PEDOT:PSS/Gr will be discussed in the following parts

**Figure 2:**
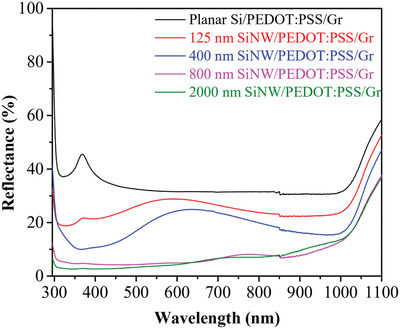
Reflectance spectra of planar Si and SiNWs coated PEDOT:PSS/Gr.

**Figure** **2** shows the effect of SiNW length on the reflectance spectra of SiNW/PEDOT:PSS/Gr in the range of 300/1100 nm. As can be seen, the sharp transition around 1000–1150 nm is attributed to the band edge of Si.^[^ ^30^ ^]^ The results show that the reflectance of SiNW is lower than planar Si (38%) and the longer SiNW structure the lower reflectance. The reflectivity of SiNW reduces from 38% to 25%, 15%, 5% corresponding to the length of SiNW: 125, 400, 800, and 2000 nm, respectively. This reduction of the reflectance could be due to the subwavelength light trapping and the light scattering interactions among the densely packed SiNWs.^[^ ^31^ ^]^ In conclusion, the SiNW structure having low reflection, strong broadband light absorption is a promising structure to improve the power conversion efficiency of the hybrid solar cells.

**Figure 3:**
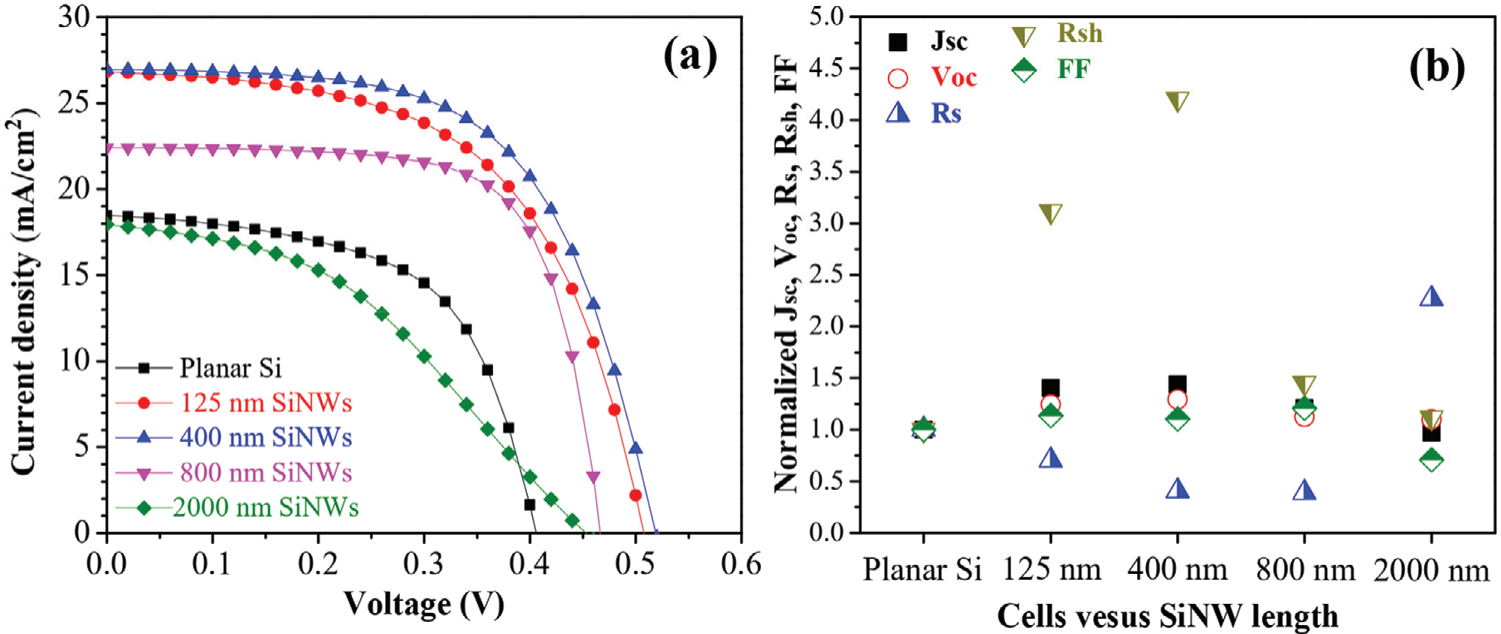
a) *J*–*V* characteristic of solar cells using different SiNW length under 100 mW cm^−2^ illumination with an AM 1.5 G solar simulator and b) solar cell parameters normalized with respect to a planar Si of hybrid solar cells based SiNWs.

The solar cell parameters such as *V*_oc_, *J*_sc_, *R*_s_, *R*_sh_, FF, and η are summarized in **Table** **1**. **Figure** **3** shows the *J*–*V* characteristic of the hybrid solar cells based on SiNW/PEDOT:PSS/Gr structures and solar cell parameters normalized with respect to a planar Si of hybrid solar cells based SiNWs. The cell using planar Si showed an η of 4.38% with a *J*_sc_ of 18.51 mA cm^−2^, a *V*_oc_ of 0.41 V, and an FF of 58%. When using SiNW as a substrate, the η increased to 8.59%, 9.05%, 7.33, and 5.32% corresponding to SiNW length of 125, 400, 800, and 2 µm, respectively. It is interesting to note that the η of the cells based SiNWs is much improved compared to that of planar Si cell. Considering the cell using 400 nm SiNWs, the η of 9.05% is nearly 2.1 times higher than that of planar Si cell (4.38%). The *J*_sc_, *V*_oc_, and FF of the cell also increased respectively to 43%, 29%, and 21% for the cell using 400 nm SiNWs. The enhancement in PCE of cells based SiNWs could be due to the low reflectance, the effective carrier transportation among SiNWs and the good carrier collection at the electrodes.^[^ ^31^ ^]^ The decrease in the reflectance is attributed to the morphology of SiNW and the presence of graphene in PEDOT:PSS solution as discussed in the previous reports.^[^ ^32^, ^33^ ^]^ Besides, the interaction between the graphene and PEDOT:PSS not only provided additional charge transport pathways in the hole transport layer but also suppressed the electron recombination at the junction interface and thus enhancing the carrier collection efficiency of the PEDOT:PSS hole transporting layer.^[^ ^32^ ^]^ The PCE of the solar cells increased with the SiNW length less than 400 nm then decreased with longer SiNWs. Solar cells based on 400 nm SiNW/PEDOT:PSS/Gr have the highest PCE of 9.05% and *J*_sc_ ≈ 26.64 mA cm^−2^, *V*_oc_ ≈ 0.53 V, FF ≈ 64%. The 400 nm SiNW solar cells having highest *R_s_*_h_ value of 626.1 2 cm^−2^ in comparison to other types of solar cells to be considered as one of the key factors lead to increasing of *V*_oc_ of the cell.^[^ ^34^ ^]^ In addition, the increase of *V*_oc_ could also result from the increase of the lifetime of minority carriers.^[^ ^34^ ^]^ Solar cells based on short SiNWs can reduce the recombination of carriers because of the short charge transport distance from p–n junction to the electrodes and thus the lifetime of minority carrier increases. The PCE of solar cells decreases with the SiNWs longer than 800 nm. This is attributed to the aggregation of SiNW tips as discussed in the previous section. This prevented the penetration of PEDOT:PSS/Gr into SiNW structure to form a large p–n junction area. Besides, the recombination in the solar cell increases when the length of SiNW increased. This is possible due to the increasing charge transport distance. **Table** **2** presents the reported performance parameters of hybrid Si/PEDOT:PSS solar cells. Comparing to other reports, the obtained results are in line with earlier reports,^[^ ^23^, ^51^, ^54^ ^]^ in which using carbon nanomaterials as an additive component for PEDOT:PSS. Gogolin et al. ^[^ ^45^ ^]^ reported a very high PCE (16.2% HV) for a hybrid solar cell, but note that the structure was used a well‐passivating electron selective a‐Si:H(i/n) layer stack at the front of our solar cells. Similarly, Fang et al. ^[^ ^46^ ^]^ reported PCE up to 12.54% as using modified Si wafer with an ideal concentration for the fabrication of the hybrid solar cells.

**Table 1:** Chemical composition and photovoltaic properties of the prepared hybrid solar cells: short circuit current density (*J*_sc_), open circuit voltage (*V*_oc_), series resistance (*R*_s_), shunt resistance (*R*_sh_), Fill factor (FF), and efficiency (η)

No.	Structure	SiNW length [nm]	*J*_sc_ [mA cm^−2^]	*V*_oc_ [V]	*R*_s_ [Ω cm^−2^]	*R*_sh_ [Ω cm^−2^]	FF [%]	η [%]
1	Planar Si/PEDOT:PSS+Gr	0	18.51	0.41	6.79	127.5	58	4.38
2	SiNWs/PEDOT:PSS+Gr	125	25.93	0.51	4.76	397.5	66	8.59
3	SiNWs/PEDOT:PSS+Gr	400	26.64	0.53	2.74	536.2	64	9.05
4	SiNWs/PEDOT:PSS+Gr	800	22.42	0.46	2.63	185.2	70	7.33
5	SiNWs/PEDOT:PSS+Gr	2000	17.95	0.45	15.39	142.5	41	5.32

**Table 2:** Reported performance parameters of hybrid Si/PEDOT:PSS solar cells

No.	Structure	Substrate	Contacts	*V*_oc_ [mV]	*J*_sc_ [mA cm^−2^]	FF [%]	η [%]	Ref.
1	SiNW/PEDOT:PSS	c‐Si	ITO	530	29.3	67	10.62	^[^ ^35^ ^]^
2	SiNH/PEDOT:PSS‐AgNW	n‐Si (100)	Ti‐Ag/ITO	574	35.55	71.37	14.56	^[^ ^36^ ^]^
3	SiNW/PEDOT:PSS	p‐Si (100)	Ni‐Ag/ITO	530	29.5	61.2	9.65	^[^ ^37^ ^]^
4	SiNW/PEDOT:PSS	n‐Si (100)	Ni‐Ag/ITO	500	29.8	58	8.7	^[^ ^38^ ^]^
5	SiNW/PEDOT:PSS‐ nc‐Si QDs	n‐Si (100)	Ti‐Ag/Ag	595	37.85	60.9	13.73	^[^ ^39^ ^]^
6	SiNW/PEDOT:PSS	n‐Si (100)	ITO	470	19.28	61	5.09	^[^ ^40^ ^]^
7	SiNW/PEDOT:PSS	n‐Si (100)	Ni‐Ag/Ag	540	18.54	65.5	6.62	^[^ ^41^ ^]^
8	SiNW/PEDOT:PSS	n‐Si (100)	Au mesh	539	36.03	67.8	13.2	^[^ ^22^ ^]^
9	SiNW/PEDOT:PSS	n‐Si (100)	Al/Cu	440	29.8	51	7.3	^[^ ^42^ ^]^
10	SiNW/PEDOT:PSS	n‐Si (100)	Al/Ag	420	24.5	41.22	4.0	^[^ ^43^ ^]^
11	SiNW/PEDOT:PSS	n‐Si (100)	Al/ITO	460	21.6	64	6.35	^[^ ^44^ ^]^
12	PEDOT:PSS/Si/a‐Si:H	p‐Si	Al/ITO	679	32	74.3	16.2	^[^ ^45^ ^]^
13	PC_61_BM/Si/PEDOT:PSS	n‐Si	Al/Ag	620	32.12	62.56	12.54	^[^ ^46^ ^]^
14	SiNW/PEDOT:PSS	n‐Si	Al/Ag	430	9.38	45	1.82	^[^ ^47^ ^]^
15	a‐Si:C/a‐Si:H/n‐Si/a‐Si:H/PEDOT:PSS	n‐Si	Ti/Al/Ag	600	30.97	59.4	11.04	^[^ ^48^ ^]^
16	SiNW/PEDOT:PSS	n‐Si (100)	Al/Cu	460	26.66	54	6.87	^[^ ^49^ ^]^
17	SiP/PEDOT:PSS	n‐Si (100)	Al/Ag	481	37.8	45.4	8.25	^[^ ^50^ ^]^
18	SiNW/PEDOT:PSS	n‐Si (100)	Al/Ag	530	26.3	64.2	9.0	^[^ ^51^ ^]^
19	Si/PEDOT:PSS	n‐Si (100)	Al/ AgNW	510	28	54	7.8	^[^ ^52^ ^]^
20	n‐Si/p‐Si/PEDOT:PSS	n‐Si (100)	Al/Ag	266	26.9	36	2.57	^[^ ^53^ ^]^
21	n+/c‐Si/PEDOT:PS‐CNT	c‐Si	Al/Ag	576	25.6	62.6	9.24	^[^ ^54^ ^]^
22	Si/PEDOT:PSS	n‐Si (100)	Ag/Ag	598	15.7	58	5.1	^[^ ^55^ ^]^
23	(n)a‐Si:H/(i)a‐Si:H/PEDOT:PSS	a‐Si	Ag/ITO	860	19.8	34	5.78	^[^ ^56^ ^]^
24	SiNWs/CuSCN/ PEDOT:PSS	n‐Si (100)	Al/Ag	610	29.2	68	12.19	^[^ ^57^ ^]^
25	SiNW/PEDOT:PSS‐GO	n‐Si (100)	Ti‐Ag/ITO	518	31	59.6	9.57	^[^ ^23^ ^]^
26	SiNW/PEDOT:PSS‐Gr	n‐Si (100)	Al/Ag	530	26.64	64	9.05	This work

In conclusion, SiNWs have shown low reflection, strong broadband light absorption, and efficient charge carrier collection across the nanowire diameter for radial junction could be considered as a promising candidate for hybrid solar cells. The PCE of SiNW/PEDOT:PSS/Gr hybrid solar cells depends on the length of SiNWs. The most suitable SiNW length is determined to be 400 nm for the fabrication of the hybrid solar cells with the power conversion efficiency measured to be 9.05% which is nearly 2.1 times higher compared to the planar Si solar cell.

## 3 Experimental Section

##### Materials

N‐type silicon wafer having a thickness of 525 µm and resistivity from 1 to 10 Ω was purchased from SEHOUNG Wafertech Company, Korea. Acetone, isopropanol, hydrofluoric (HF) acid, H_2_SO_4_ (98%), HNO_3_ (68%), H_2_O_2_, KOH, AgNO_3_, were purchased from Shantou Xilong Chemical Company, China.

##### Fabrication of Silicon Nanowires

Clean n‐type Si was cut into pieces of 1.5 × 1.5 cm in size. These pieces Si substrates were dipped in HF solution (2%) for 2 min to remove the SiO_2_ layer on top of the Si surface. The SiNW structure was formed using a one‐step chemical wet etching process. The clean Si substrates were immersed in etching solution which was a mixture of HF (4.6 m) and AgNO_3_ (0.02 m) for different times of 1, 3, 6, …, 60 min. After finishing the etching process, the Si substrates were immersed in deionized (DI) water to remove all residue acid, followed by HNO_3_/H_2_O (1/1) solution in order to remove all residual Ag that was formed during the etching process, which was followed by washing with DI and dried under N_2_ gas flow.

##### Fabrication of Solar Cells

Firstly, graphene was functionalized using a mixture of acid H_2_SO_4_:HNO_3_ (3:1) at 70 °C for 5 h to attach the carboxylic (COOH) functional group on the surface. The functionalized Gr was dispersed into deionized water with a concentration of 1 mg mL^−1^ by ultrasonic for 45 min at room temperature. Then the functionalized Gr solution was dispersed into PEDOT:PSS with a concentration of 0.5% in weight by ultrasonic for 6 h to obtain a homogenous PEDOT:PSS/Gr solution. Secondly, the PEDOT:PSS/Gr solution was coated on the surface of SiNWs by a spin coating method, following these spin steps: 2000 rpm during 10 s then 6000 rpm during 60 s. After that, the n‐SiNW/PEDOT:PSS/Gr was baked in an oven with N_2_ gas environment at 140 °C for 30 min. Finally, a finger‐typed 250 nm Ag film and 250 nm Al were deposited on the top of the structure and the backside to form a front and rear electrode electrodes using the sputtering method.

##### Characterization

The surface morphology of the samples was studied by field emission scanning electron microscope (FESEM, Hitachi S4800). UV–vis spectroscopy was used to measure the optical reflectance spectra of samples by using a Jacob V‐570 UV/vis/NIR spectrophotometer. The *J*–*V* characteristic of solar cells was measured by Keithley 2400 under an illumination condition of AM 1.5 G with a density of 100 mW cm^−2^

## Conflict of Interest

The authors declare no conflict of interest.
